# Newly Diagnosed and Growing Subependymal Giant Cell Astrocytoma in Adults With Tuberous Sclerosis Complex: Results From the International TOSCA Study

**DOI:** 10.3389/fneur.2019.00821

**Published:** 2019-08-02

**Authors:** Anna C. Jansen, Elena Belousova, Mirjana P. Benedik, Tom Carter, Vincent Cottin, Paolo Curatolo, Lisa D'Amato, Guillaume Beaure d'Augères, Petrus J. de Vries, José C. Ferreira, Martha Feucht, Carla Fladrowski, Christoph Hertzberg, Sergiusz Jozwiak, John A. Lawson, Alfons Macaya, Ruben Marques, Rima Nabbout, Finbar O'Callaghan, Jiong Qin, Valentin Sander, Matthias Sauter, Seema Shah, Yukitoshi Takahashi, Renaud Touraine, Sotiris Youroukos, Bernard Zonnenberg, John C. Kingswood

**Affiliations:** ^1^Pediatric Neurology Unit, Department of Pediatrics, UZ Brussel VUB, Brussels, Belgium; ^2^Research and Clinical Institute of Pediatrics, Pirogov Russian National Research Medical University, Moscow, Russia; ^3^Child Neurology Department, SPS Pediatrična Klinika, Ljubljana, Slovenia; ^4^Tuberous Sclerosis Association (TSA), Nottingham, United Kingdom; ^5^Hôpital Louis Pradel, Claude Bernard University Lyon 1, Lyon, France; ^6^Child Neurology and Psychiatry Unit, Systems Medicine Department, Tor Vergata University Hospital, Rome, Italy; ^7^Novartis Farma S.p.A., Origgio, Italy; ^8^Association Sclérose Tubéreuse de Bourneville, Gradignan, France; ^9^Division of Child and Adolescent Psychiatry, University of Cape Town, Cape Town, South Africa; ^10^Neurology Department, Centro Hospitalar Lisboa Ocidental, Lisbon, Portugal; ^11^Medical University of Vienna, Universitätsklinik für Kinder-und Jugendheilkunde, Vienna, Austria; ^12^Associazione Sclerosi Tuberosa ONLUS, Milan, Italy; ^13^European Tuberous Sclerosis Complex Association, Dattein, Germany; ^14^Zentrum für Neuropädiatrie und Sozialpädiatrie, Vivantes-Klinikum Neukölln, Berlin, Germany; ^15^Department of Child Neurology, Warsaw Medical University, Warsaw, Poland; ^16^Department of Neurology and Epileptology, The Children's Memorial Health Institute, Warsaw, Poland; ^17^The Tuberous Sclerosis Multidisciplinary Management Clinic, Sydney Children's Hospital, Randwick, NSW, Australia; ^18^Pediatric Neurology Section, Hospital Universitari Vall d'Hebron, Barcelona, Spain; ^19^Institute of Biomedicine (IBIOMED), University of Leon, León, Spain; ^20^Department of Pediatric Neurology, Necker Enfants Malades Hospital, Paris Descartes University, Paris, France; ^21^Paediatric Neuroscience, Institute of Child Health, University College London, London, United Kingdom; ^22^Department of Pediatrics, Peking University People's Hospital, Beijing, China; ^23^Neurology and Rehabilitation, Tallinn Children Hospital, Tallinn, Estonia; ^24^Klinikum Kempten, Klinikverbund Kempten-Oberallgäu gGmbH, Kempten, Germany; ^25^Novartis Healthcare Pvt Ltd., Hyderabad, India; ^26^National Epilepsy Center, Shizuoka Institute of Epilepsy and Neurological Disorders, NHO, Aoi-ku, Japan; ^27^Department of Genetics, CHU-Hôpital Nord, Saint Etienne, France; ^28^First Department of Paediatrics, Athens University, St. Sophia Children's Hospital, Athens, Greece; ^29^Department of Internal Medicine, University Medical Center, Utrecht, Netherlands; ^30^Cardiology Clinical Academic Group, Molecular and Clinical Sciences Research Centre, St. Georges University of London, London, United Kingdom

**Keywords:** mTOR, registry, SEGA, TOSCA, tuberous sclerosis complex

## Abstract

The onset and growth of subependymal giant cell astrocytoma (SEGA) in tuberous sclerosis complex (TSC) typically occurs in childhood. There is minimal information on SEGA evolution in adults with TSC. Of 2,211 patients enrolled in TOSCA, 220 of the 803 adults (27.4%) ever had a SEGA. Of 186 patients with SEGA still ongoing in adulthood, 153 (82.3%) remained asymptomatic, and 33 (17.7%) were reported to ever have developed symptoms related to SEGA growth. SEGA growth since the previous scan was reported in 39 of the 186 adults (21%) with ongoing SEGA. All but one patient with growing SEGA had mutations in *TSC2*. Fourteen adults (2.4%) were newly diagnosed with SEGA during follow-up, and majority had mutations in *TSC2*. Our findings suggest that surveillance for new or growing SEGA is warranted also in adulthood, particularly in patients with mutations in *TSC2*.

## Introduction

Tuberous sclerosis complex (TSC) is an autosomal dominant disorder characterised by hamartomas in multiple organs, with the brain being the most commonly affected organ (1, 2). Subependymal giant cell astrocytoma (SEGA) occurs at the foramen of Monro, with a reported lifetime prevalence between 5 and 24% (3, 4). Although SEGAs are generally benign and non-infiltrative, these may grow, and obstruct cerebrospinal fluid (CSF) flow, thereby increasing intracranial pressure. Typical symptoms of growing SEGA include headaches, blurred vision, nausea, vomiting, worsening of seizure control or new-onset seizures, and sudden death from acute hydrocephalus (3, 5).

Diagnosis of SEGA has changed from pathology-based to imaging-based (6, 7), but formal diagnostic criteria have only been available since 2012, when an expert panel at the International Tuberous Sclerosis Complex Consensus Conference defined SEGA as a lesion at the caudothalamic groove with a size of >1 cm in any direction or a subependymal lesion at any location which has shown serial growth on consecutive imaging regardless of size (7). All SEGA-related studies performed before 2012 have been based on variable criteria, thus limiting the value of comparison (8).

Onset and growth of SEGA has been reported most commonly in the first two decades of life (9). In two of the largest series of operated SEGAs, the mean age of surgical intervention was 9.7 years (10), and 11.6 years, (11) suggesting that growth is most common at this age. SEGA have been reported in neonates (9). Data on SEGA prevalence and growth in adults are scarce. A retrospective case series of 16 patients with TSC who required SEGA surgery, highlighted that SEGA can still become symptomatic later in life (12).

Present guidelines recommend that patients with asymptomatic SEGA diagnosed during childhood should continue to be imaged periodically as adults to ensure that there is no growth (13). Patients with large or growing SEGA or with SEGA causing ventricular enlargement that are still asymptomatic, should undergo MRI (magnetic resonance imaging) scans more frequently, and such patients and their families should be educated regarding the symptoms of raised intracranial pressure (7).

Surgical resection (occasionally VP shunt alone) is the recommended intervention for acutely symptomatic individuals, while either surgical resection or medical therapy with mammalian/mechanistic target of rapamycin (mTOR) inhibitors can be effective for individuals with growing asymptomatic SEGA (13). Treatment decisions should be based on multiple factors such as the patient's clinical condition, anatomic considerations specific to SEGA, surgeon's experience, experience of the centre regarding use of mTOR inhibitors, prior history of SEGA resection, other TSC-related comorbidities, and patient/parental preference (7).

This is the first study evaluating prevalence, growth, symptoms, and treatment patterns in a large prospective cohort of adults with TSC-associated SEGA.

## Methods

TOSCA, a large-scale non-interventional study in patients with TSC, was conducted at 170 sites in 31 countries. The study design and methodology of TOSCA has been published previously (14). The study enrolled patients of any age with TSC between August 2012 and November 2014 and followed for up to 5 years. Patient data, including demographics, and information related to clinical features of TSC across all organ systems, comorbidities and rare manifestations, were collected at baseline and at regular visits scheduled at a maximum interval of 1 year.

In this study, designed prior to the 2012 imaging-based consensus, prevalence, and growth of SEGA were defined as per clinical practice of the participating centres. We evaluated SEGA manifestations among adult patients (>18 years) enrolled into the TOSCA study. SEGA-related questions included in the case report form (CRF) were presence of single or multiple SEGA, newly diagnosed SEGA, SEGA growth, clinical signs, and symptoms associated with SEGA and information regarding SEGA treatment. In addition, possible associations of SEGA prevalence with genotype were analysed using a Chi-square test. Statistical significance was set at *p*-value < 0.05.

Statistics were descriptive considering the exploratory nature of this study. Categorical data were reported as frequencies and percentages, and continuous variables were expressed as mean (± standard deviation) or as median (range), unless stated otherwise.

The study was designed and conducted in accordance with the Good Clinical Practice principles, the Declaration of Helsinki and all local regulations. The institutional review board or ethics committee at each participating site approved required TOSCA-related documents. Written informed consent was obtained from all patients, parents or guardians before enrolment.

## Results

A total of 2,214 patients with TSC were enrolled in TOSCA study, and data were analysed for 2,211 patients. In the final analysis performed on data collected until August 2017, a history of SEGA was reported in 30.3% (671/2,211; 332 males and 339 females) of patients. Other neuroimaging features reported included cerebral white matter radial migration lines in 25.5, cortical tubers in 87.2, and subependymal nodules 82.9%.

Of the 803 adult patients included in the final analysis, a history of SEGA was reported in 220 patients (27.4%). The demographic of the adult patients with SEGA are shown in Table 1. SEGA were ongoing during study in 186 (84.5%) patients. Of these, multiple and bilateral SEGA were reported in 66 (35.5%), and 61 (32.8%) patients, respectively. SEGA growth since previous scan was reported in 39 (21%). The median age at SEGA diagnosis in this adult cohort was 20 years (range, <1–57 years), as compared to 7 years (range, <1–57 years) in the entire TOSCA cohort.

**Table 1:** Demographics of adult patients with SEGA.

**Characteristics**	**Patients with SEGA**			
	**All adults (*n* = 220)**	**>18 to ≤25 years** **(*n* = 91)**	**>25 to ≤40 years** **(*n* = 96)**	**>40 years** **(*n* = 33)**
Age at diagnosis of TSC, years; median (range)	4.0 (<1–48)	1.0 (<1–24)	4.0 (<1–37)	15.0 (<1–48)
**Gender**, ***n*** **(%)**				
Male	98 (44.5)	35 (38.5)	46 (47.9)	17 (51.5)
Female	122 (55.5)	56 (61.5)	50 (52.1)	16 (48.5)
Patients with molecular testing, *n* (%)	96 (43.6)	40 (44.0)	41 (42.7)	15 (45.5)
**Genetic Testing**, ***n*** **(%)**				
No mutation identified	12 (12.5)	6 (15.0)	3 (7.3)	3 (20.0)
*TSC1* mutation	12 (12.5)	2 (5.0)	5 (12.2)	5 (33.3)
*TSC2* mutation	69 (71.9)	31 (77.5)	31 (75.6)	7 (46.7)
Results not available^*^	5 (5.2)	1 (2.5)	1 (2.4)	0
**Variation Type**, ***n*** **(%)**				
Pathogenic mutation	59 (61.5)	22 (55.0)	27 (65.9)	10 (66.7)
Variant of unknown significance	5 (5.2)	4 (10.0)	1 (2.4)	0
Both pathogenic mutation and variant of unknown significance	2 (2.1)	0	2 (4.9)	0
Results not available^*^	30 (31.3)	14 (35)	11 (26.8)	5 (33.3)
Patients with prenatal diagnosis, *n* (%)	1 (0.5)	1 (1.1)	0	0

*Values are expressed as n (%), unless otherwise specified*.

* *Include missing data and those results not made available due to legal/medical confidentiality statements*.

*SEGA, subependymal giant cell astrocytoma. TSC, tuberous sclerosis complex*.

The median interval between consecutive scans was 1 year (range <1–34 years). During the study period (up to 5 years), 14 new diagnoses of SEGA were made (2.4% of total adults minus those with history of SEGA). The oldest patient with a newly reported SEGA was 57 years. Of the 186 adults with ongoing SEGA, 153 (82.3%) remained asymptomatic, and 33 (17.7%) were reported to ever have developed symptoms related to SEGA growth in the past, including primarily increase in seizure frequency (15.6%), behavioural disturbance (13.4%), and headache (10.8%), either alone or in combination with other symptoms (Table 2). Over time, SEGA had been treated with surgery in 55 out of 117 patients (47.0%) and with mTOR-inhibitors in 46 out of 117 patients (39.3%). Nine patients (7.7%) required a shunt for the management of hydrocephalus.

**Table 2:** Clinical characteristics of SEGA.

	**Overall TOSCA population (*n* = 2211)**	**Adult patients**			
		**All adults** **(*n* = 803)**	**>18 to ≤25 years** **(*n* = 235)**	**>25 to ≤40 years** **(*n* = 344)**	**>40 years** **(*n* = 224)**
Patients with history of SEGA	671 (30.3)	220 (27.4)	91 (38.7)	96 (27.9)	33 (14.7)
No. of patients with ongoing SEGA during the study, *n*	579	186	71	87	28
Multiple	240 (41.5)	66 (35.5)	24 (33.8)	33 (37.9)	9 (32.1)
Bilateral	236 (40.8)	61 (32.8)	21 (29.6)	30 (34.5)	10 (35.7)
Growing SEGA since previous scan^*^^#^	208 (35.9)	39 (21.0)	19 (26.8)	17 (19.5)	3 (10.7)
**Signs and symptoms**					
None	476 (82.2)	153 (82.3)	57 (80.3)	72 (82.8)	24 (85.7)
Increase in seizure frequency	98 (16.9)	29 (15.6)	14 (19.7)	13 (14.9)	2 (7.1)
Behavioural disturbance	77 (13.3)	25 (13.4)	8 (11.3)	16 (18.4)	1 (3.6)
Regression/loss of cognitive skills	51 (8.8)	16 (8.6)	5 (7.0)	10 (11.5)	1 (3.6)
Headache	47 (8.1)	20 (10.8)	7 (9.9)	10 (11.5)	3 (10.7)
Ventriculomegaly	32 (5.5)	8 (4.3)	5 (7.0)	3 (3.4)	0
Increased intracranial pressure	24 (4.1)	10 (5.4)	6 (8.5)	2 (2.3)	2 (7.1)
Sleep disorder	22 (3.8)	7 (3.8)	1 (1.4)	6 (6.9)	0
Eye movement abnormalities	16 (2.8)	6 (3.2)	4 (5.6)	2 (2.3)	0
Visual impairment	10 (1.7)	4 (2.2)	3 (4.2)	1 (1.1)	0
Papilloedema	8 (1.4)	4 (2.2)	2 (2.8)	1 (1.1)	1 (3.6)
Neuroendocrine dysfunction	8 (1.4)	4 (2.2)	0	3 (3.4)	1 (3.6)
Other	28 (4.8)	7 (3.8)	4 (5.6)	3 (3.4)	0

*Values are expressed as n (%), unless otherwise specified*.

* *Median time from previous scan to last assessment was 1 year*.

# *Growing of SEGA since previous scan was measured among those with ongoing SEGA during the study. SEGA, subependymal giant cell astrocytoma*.

SEGA were significantly more frequent in adults with a *TSC2* mutation compared to those with a *TSC1* mutation (35.2 vs. 15.6%, *p* < 0.0004). However, there was no significant difference in multiple (*p* = 0.1158), bilateral (*p* = 0.1062), or growing SEGA (*p* = 1.0000), and presence of SEGA-related symptoms (*p* = 0.2598) between those with *TSC1* and *TSC2* mutation. The median age at SEGA diagnosis was higher in patients with *TSC1* mutations (29 years, range 9–51) compared to patients with *TSC2* mutations (21 years, range <1–49), but this difference was non-significant (Table 3). Furthermore, 12 of 14 adults with newly diagnosed SEGA had mutations in *TSC2* gene, while two had no mutation identified.

**Table 3:** Clinical characteristics of SEGA in adults with mutations in *TSC1* vs. *TSC2*.

	**Adults with *TSC1* mutation (*n* = 77)**	**Adults with *TSC2* mutation (*n* = 196)**	***p*-value**
Patients with history of SEGA	12 (15.6)	69 (35.2)	0.0004
Median (range) age at SEGA diagnosis, years	29 (9–51)	21 (<1–49)	0.0599
No. of patients with ongoing SEGA during the study	8 (66.7)	61 (88.4)	0.1317
Multiple	5 (62.5)	19 (31.1)	0.1158
Bilateral	5 (62.5)	18 (29.5)	0.1062
Growing SEGA since previous scan	1 (12.5)	13 (21.3)	1.0000
**Signs and Symptoms**			
None	5 (62.5)	49 (87.5)	0.3580
Increase in seizure frequency	3 (37.5)	15 (28.3)	0.6243
Behavioural disturbance	1 (12.5)	14 (26.4)	1.0000
Headache	1 (12.5)	10 (18.9)	0.5753
Regression/loss of cognitive skills	0	5 (9.4)	1.0000
Ventriculomegaly	0	4 (7.5)	1.0000
Increased intracranial pressure	1 (12.5)	3 (5.7)	1.0000
Papilloedema	1 (12.5)	3 (5.7)	1.0000
Sleep disorder	0	2 (3.8)	1.0000
Eye movement abnormalities	0	2 (3.8)	1.0000
Visual impairment	0	2 (3.8)	1.0000
Neuroendocrine dysfunction	1 (12.5)	2 (3.8)	0.2408
Other	1 (12.5)	3 (5.7)	0.3098
Patients received treatment	8 (66.7)	37 (53.6)	0.0716

*Values are expressed as n (%), unless otherwise specified*.

*SEGA, subependymal giant cell astrocytoma*.

## Discussion

To our knowledge, this is the first study to evaluate SEGA prevalence, growth, symptoms, and current treatment modalities in adults with TSC-associated SEGA. The international TOSCA study allowed us to evaluate data from 803 adults (age > 18 years), 220 of whom had SEGA (27.4%). During the 5 years follow-up period of the study, 23.2% of adults reported that the SEGA was still ongoing.

The occurrence of new SEGA after the age of 18 years was relatively low (2.4%) but more common than previously thought (7). In this cohort, age at SEGA diagnosis was as late as 57 years. Newly diagnosed SEGA were associated with mutations in *TSC2* in the large majority of cases (85.7%). Other risk factors such as contrast enhancement of SEN in the caudo-thalamic groove were beyond the scope of this study.

Another key finding was that SEGA growth since previous scan (mean time of 1.5–2.3 years between previous scan and last assessment) was observed in 21% of our adult patients. Although not negligible, this is less frequent compared with children. In a cohort of 58 patients (33 children, 25 adults), Tsai et al. reported similar results, with SEGA growth in children being significantly higher than in adults (75.6 vs. 16.5%) (15).

The fact that SEGA may still grow during adulthood emphasises the need for continuous surveillance even after the age of 25 years. This was highlighted in the current guidelines that recommend that patients with asymptomatic SEGA diagnosed in childhood should continue to undergo periodical imaging as adults to ensure that there is no growth. This highlights the need for continued multidisciplinary follow-up, also at adult age. Although newly occurring SEGA during adulthood seem relatively rare and do not warrant systematic screening, physicians should keep this possibility in mind when symptoms potentially related to SEGA growth occur. Special attention should be paid to adults with mutations in *TSC2* since they seem to be at a higher risk for newly occurring SEGA and SEGA growth in adulthood as well as to individuals with intellectual disability who might not be able to verbally express SEGA-related symptoms. Importantly, certain SEGA-related symptoms (especially early symptoms) are not limited to signs of increased intracranial pressure, and therefore, parents and patients should be informed about all relevant symptoms which require referral for medical evaluation, particularly sudden behavioural changes such as acute-onset and unexplained aggression, academic difficulties or any other acute and unexplained manifestations of TSC-associated neuropsychiatric disorders (TAND) (16–18).

We acknowledge the limitations intrinsic to a large-scale, international, non-interventional/observational study. These included the fact that participants were recruited from expert TSC centres around the world and the fact that data on SEGA diagnosis, growth and SEGA-related symptoms were collected as reported per clinical practice. However, these limitations are, at least in part, offset by the large-scale and “real-world” nature of the cohort across multiple centres and countries. Being an observational study, detailed information on the treatment initiated for SEGA at adult age were not collected. The very low number of missing data for SEGA reflects good quality of data collection for this specific manifestation.

## Conclusion

Findings from this large international study highlight the need for continued monitoring for SEGA growth in adults with ongoing SEGA. Clinicians and adults with TSC should be aware of the potential new onset SEGA in adults with SEGA-related symptoms, especially in the presence of mutations in *TSC2*.

## Data Availability

Novartis supports publication of scientifically rigorous analysis which is relevant to patient care, regardless of a positive or negative outcome. Qualified external researchers can request access to anonymised patient-level data, respecting patient informed consent, by contacting study sponsor authors. The protocol can be accessed through the EnCePP portal http://www.encepp.eu/ (EU PAS Register Number EUPAS3247).

## Ethics Statement

The study protocol and all amendments were reviewed and approved (if applicable) by independent ethics committee/institutional review board for each centre: National Hospital Organization Central Ethics Committee; Gazi University Clinical Research Ethics Committee; Independent Multidisciplinary Committee on Ethical Review of Clinical Trials; Peking Union Medical College Hospital; Commissie Medische Ethiek UZ Brussel; CNIL (Commission National de l'Informatique et des Libertés), CCTIRS (Comité Consultatif sur le traitement de l'information en matière de recherche dans le domaine de la santé); CEIC-E (Comité Etico Investigación Clínica de Euskadi; Consejeria de Salud y Bienestar Social, Dirección General de Calidad, Investigación, Desarrollo e Innovación, Comité Coordinador de Ética de la Investigación Biomédica de Andalucía; UT REC (Research Ethics Committee of the University of Tartu); Ethikkommission der Medizinischen Universität Graz; North Wales REC–West; Regionala Etikprövningsnämnden i Göteborg; REK–Regionale komiteer for medisinsk og helsefaglig forskningsetikk; Komisja Bioetyczna przy Instytucie Pomnik Centrum Zdrowia Dziecka; Ethikkommission bei der Ludwig-Maximilians-Universitat München; Hokkaido University Hospital Independent clinical research Institutional Ethics Committee; Medical Juntendo University Institutional Ethics Committee; National Center for Chile Health and Development of IRB; Osaka University Hospital of IRB; Ethics Committee at Moscow Institute of Pediatrics and Pediatric Surgery; Peking University First Hospital; Sanbo Brain Hospital Capital Medical University; Tianjin Children's Hospital; Childrens Hospital Of Fudan University; Zhongshan Hospital Fudan University; Fudan University Shanghai Cancer Center; The Second Affiliated Hospital of Guangzhou Medical University; The First Affiliated Hospital, Sun Yan-Sen University; The First Affiliated Hospital of Guangzhou Medical University; Shenzhen Children's Hospital; West China Hospital, Sichuan University; Xijing Hospital; Children's Hospital of Chongqing Medical University; Wuhan Children's Hospital; The second affiliated hospital of Xi'an jiaotong university; Guangdong 999 brain hospital; Seoul National University Hospital Institutional Review Board; National Taiwan University Hospital (NTUH) Research Ethics Committee (REC); Institutional Review Board of the Taichung Veterans General Hospital; Institutional Review Board of Chung Shan Medical University Hospital; Institutional Review Board, Tungs' Taichung MetroHarbor Hospital; Institutional Review Board of National Cheng Kung University Hospital; Metro South Human Research Ethics Committee; Sydney Children's Hospital Network Human Research Ethics Committee; St Vincents Hospital Human Research Ethics Committee; Royal Melbourne Hospital Human Research Ethics Committee; Siriraj Institutional Review Board; The Institutional Review board, Faculty of Medicine, Chulalongkorn University, 3rd Floor, Ananthamahidol Building, King Chulalongkorn Memorial Hospital; The committee on Human Rights Related to Research Involving Human Subjects; Institutional Review board, Royal Thai Army Medical Department IRB RTA, 5th Floor, Phramongkutklaowejvitya Building, Phramongkutklao College of Medicine; Research Ethics Committee, Faculty of Medicine, Chiang Mai University; Research and Development, Queen Sirikit National Institute of Child Health; Human Research Ethics Committee, Faculty of Health Sciences, University of Cape Town; Shaare Zedek Medical center Helsinki committee; Sheba Medical center Helsinki committee; Tel Aviv Sourasly Medical center Helsinki committee; General University Hospital of Patras Ethics Committee; Pendeli Children's Hospital Ethics Committee; General University Hospital of Athens G. Gennimatas Ethics Committee; Evaggelismos General Hospital Ethics Committee; General University Hospital of Thessaloniki AHEPA Ethics Committee; General University Hospital of Ionnina Ethics Committee; METC UMC Utrecht; Direcció General de Regulació, Planificació i Recursos Sanitaris; Comité Ético de Investigación Clínica del Hospital Universitario Vall d'Hebron de Barcelona, Generalitat de Catalunya, Departament de Salut; Comité Ético de Investigación Clínica Hospital Universitario La Paz; Dirección General de Ordenación e Inspección, Consejería de Sanidad Comunidad de Madrid, Servicios de Control Farmacéutico y Productos Sanitarios; Comité Etico Investigación Clínica del Hospital Universitario y Politécnico de La Fe; Dirección General de Farmàcia i Productes Sanitaris, Generalitat de Valencia; Comité de Ética de la Investigación de Centro de Granada; IACS (Instituto Aragonés de Ciencias de la Salud); Comité Etico Investigación Clínica Regional del Principado de Asturias; Comité Etico Investigación Clínica Hospital 12 de Octubre; Comité Etico Investigación Clínica Hospital Universitario Virgen de la Arrixaca; Sección de Ordenación e Inspección Farmacéutica Departamento de Salud; Comité Ético de Investigación Clínica del Hospital Universitario del Río Hortega de Valladolid; CES (Comissão de Ética para a Saúde), Centro Hospitalar de Lisboa Ocidental, EPE; CES (Comissão de Ética para a Saúde), Centro Hospitalar do Porto, E.P.E; CES (Comissão de Ética para a Saúde), Centro Hospitalar Lisboa Central, EPE; CES (Comissão de Ética para a Saúde), Hospital Garcia de Orta, EPE; CES (Comissão de Ética para a Saúde), Centro Hospitalar de São João, EPE; CES (Comissão de Ética para a Saúde), Hospital Professor Doutor Fernando Fonseca, EPE; CES (Comissão de Ética para a Saúde), Centro Hospitalar do Algarve, EPE (Unidade de Faro); LUHS Kaunas Regional Biomedical Research Ethics Committee; Paula Stradina kliniskās universitātes slimnicas, Attistibas biedribas Kliniskās izpētes Etikas komiteja, Ethics Committee for Clinical Research; Komisija Republike Slovenije za medicinsko etiko; Comitato Etico Indipendente Presso La Fondazione Ptv Policlinico Tor Vergata Di Roma; Comitato Etico Regione Calabria Sezione Centro c/o A.O.U. Mater Domini Di Catanzaro; Comitato Etico Azienda Ospedaliera Universitaria Di Cagliari; Comitato Etico Cardarelli-Santobono c/o Ao Cardarelli; Comitato Etico Per La Sperimentazione Clinica Delle Province Di Verona E Rovigo, Presso Aoui Verona; Eticka Komise Fn Brno; Eticka Komisia Dfnsp Bratislava; Eticka Komisia Pri Dfn Kosice; Eticka Komisia Bratislavskeho Samospravneho Kraja; Comisia Naţional*ă* de Bioetic*ă* a Medicamentului şi a Dispozitivelor Medicale; Comitato Etico Milano area 1 c/o ASST FBF Sacco-P.O. L. Sacco; Comité de Ética de la Investigación de Centro Hospital Universitario Virgen del Rocío; Comité Ético de Investigación Clínica Fundació Sant Joan de Déu Generalitat de Catalunya. Departament de Salut; Comité Ético de Investigación Clínica Hospital Infantil Universitario Niño Jesús; Consejería de Sanidad Dirección General de Salus Pública Junta de Castilla León; Dirección General de Asistencia Sanitaria, Consejería de Sanidad Gobierno del Principado de Asturias; Dirección General de Planificación, Ordenación Sanitaria y Farmacéutica e Investigación, Consejeria de Sanidad y Política Social Región de Murcia; Ethics Committee at Moscow Institute of Pediatrics and Pediatric Surgery; Paula Stradina kliniskās universitātes slimnicas, Attistibas biedribas Kliniskās izpētes Etikas komiteja, Ethics Committee for Clinical Research; The First Affiliated Hospital of The Fourth Military Medical University; Zhongshan hospital Fudan University.

## Author Contributions

AJ, EB, MB, PC, JF, MF, CH, SJ, JL, AM, RN, VS, MS, RT, BZ, and JK: designing the study, patient accrual, clinical care, data interpretation, drafting, revising, final review, and approval of the manuscript. TC, VC, LD'A, GBA, PV, CF, FO'C, JQ, YT, and SY: designing the study, trial management, data collection, data analysis, data interpretation, drafting, revising, final review, and approval of the manuscript. RM: designing the study, data analysis, data interpretation, drafting, revising, final review, and approval of the manuscript. SS: designing the study, trial statistician, data analysis, data interpretation, drafting, revising, final review, and approval of the manuscript.

### Conflict of Interest Statement

AJ, EB, TC, VC, PC, GBA, PV, JK, JF, MF, CF, CH, SJ, RN, FO'C, JQ, MS, RT, JL, AM, SY, MB, and BZ received honoraria and support for travel from Novartis. VC received personal fees for consulting, lecture fees, and travel from Actelion, Bayer, Biogen Idec, Boehringer Ingelheim, Gilead, GSK, MSD, Novartis, Pfizer, Roche, and Sanofi; grants from Actelion, Boehringer Ingelheim, GSK, Pfizer, and Roche; personal fees for developing educational material from Boehringer Ingelheim and Roche. PV has been on the study steering group of the EXIST-1, 2, and 3 studies sponsored by Novartis and co-PI on two investigator-initiated studies part-funded by Novartis. RN received grant support, paid to her institution, from Eisai and lectures fees from Nutricia, Eisai, Advicenne, and GW Pharma. YT received personal fee from Novartis for lecture and for copyright of referential figures from the journals and received grant from Japanese government for intractable epilepsy research. SJ was partly financed by the EC Seventh Framework Programme (FP7/2007-2013; EPISTOP, grant agreement no. 602391), the Polish Ministerial funds for science (years 2013–2018) for implementation of international co-financed project and the grant EPIMARKER of the Polish National Center for Research and Development No STRATEGMED3/306306/4/2016. JK, PC, CH, JL, and JQ received research grant from Novartis. RM and SS are employees of Novartis, while LD'A was a Novartis employee at the time of manuscript concept approval. This study was funded by Novartis Pharma AG. All authors approved the final version of the manuscript prior to submission.

## TOSCA Investigators

Japan: Nobuo Shinohara, Shigeo Horie, Masaya Kubota, Jun Tohyama, Katsumi Imai, Mari Kaneda, Hideo Kaneko, Yasushi Uchida, Tomoko Kirino, Shoichi Endo, Yoshikazu Inoue, Katsuhisa Uruno; Turkey: Ayse Serdaroglu, Zuhal Yapici, Banu Anlar, Sakir Altunbasak; Russia: Olga Lvova, Oleg Valeryevich Belyaev, Oleg Agranovich, Elena Vladislavovna Levitina, Yulia Vladimirovna Maksimova, Antonina Karas; China: Yuwu Jiang, Liping Zou, Kaifeng Xu, Yushi Zhang, Guoming Luan, Yuqin Zhang, Yi Wang, Meiling Jin, Dingwei Ye, Weiping Liao, Liemin Zhou, Jie Liu, Jianxiang Liao, Bo YAN, Yanchun Deng, Li Jiang, Zhisheng Liu, Shaoping Huang, Hua Li; Korea: Kijoong Kim; Taiwan: Pei-Lung Chen, Hsiu-Fen Lee, Jeng-Dau Tsai, Ching-Shiang Chi, Chao-Ching Huang; Australia: Kate Riney, Deborah Yates, Patrick Kwan; Thailand: Surachai Likasitwattanakul, Charcrin Nabangchang, Lunliya Thampratankul Krisnachai Chomtho, Kamornwan Katanyuwong, Somjit Sriudomkajorn; South Africa: Jo Wilmshurst; Israel: Reeval Segel, Tal Gilboa, Michal Tzadok, Aviva Fattal- Valevski; Greece: Panagiotis Papathanasopoulos, Antigone Syrigou Papavasiliou, Stylianos Giannakodimos, Stylianos Gatzonis, Evangelos Pavlou, Meropi Tzoufi; Netherlands: A.M.H. Vergeer; Belgium: Marc Dhooghe, Hélène Verhelst, Filip Roelens, Marie Cecile Nassogne, Pierre Defresne, Liesbeth De Waele, Patricia Leroy, Nathalie Demonceau, Benjamin Legros, Patrick Van Bogaert, Berten Ceulemans, Lina Dom; France: Pierre Castelnau, Anne De Saint Martin, Audrey Riquet, Mathieu Milh, Claude Cances, Jean-Michel Pedespan, Dorothee Ville, Agathe Roubertie, Stéphane Auvin, Patrick Berquin, Christian Richelme, Catherine Allaire, Sophie Gueden, Sylvie Nguyen The Tich, Bertrand Godet; Spain: Maria Luz Ruiz Falco Rojas, Jaume Campistol Planas, Antonio Martinez Bermejo, Patricia Smeyers Dura, Susana Roldan Aparicio, Maria Jesus Martinez Gonzalez, Javier Lopez Pison, Manuel Oscar Blanco Barca, Eduardo Lopez Laso, Olga Alonso Luengo, Francisco Javier Aguirre Rodriguez, Ignacio Malaga Dieguez, Ana Camacho Salas, Itxaso Marti Carrera, Eduardo Martinez Salcedo, Maria Eugenia Yoldi Petri, Ramon Cancho Candela; Portugal: Ines da Conceicao Carrilho, Jose Pedro Vieira, José Paulo da Silva Oliveira Monteiro, Miguel Jorge Santos de Oliveira Ferreira Leao, Catarina Sofia Marceano Ribeiro Luis, Carla Pires Mendonca; Lithuania: Milda Endziniene; Latvia: Jurgis Strautmanis; Estonia: Inga Talvik; Italy: Maria Paola Canevini, Antonio Gambardella, Dario Pruna, Salvatore Buono, Elena Fontana, Bernardo Dalla Bernardina; Romania: Carmen Burloiu, Iuliu Stefan Bacos Cosma, Mihaela Adela Vintan, Laura Popescu; Czech Republic: Karel Zitterbart; Slovakia: Jaroslava Payerova, Ladislav Bratsky, Zuzana Zilinska; Austria: Ursula Gruber-Sedlmayr, Matthias Baumann, Edda Haberlandt, Kevin Rostasy, Ekaterina Pataraia; United Kingdom: Frances Elmslie, Clare Ann Johnston, Pamela Crawford; Denmark: Peter Uldall; Sweden: Maria Dahlin, Paul Uvebrant, Olof Rask; Norway: Marit Bjoernvold, Eylert Brodtkorb, Andreas Sloerdahl, Ragnar Solhoff, Martine Sofie Gilje Jaatun; Poland: Marek Mandera, Elzbieta Janina Radzikowska, Mariusz Wysocki; Germany: Michael Fischereder, Gerhard Kurlemann, Bernd Wilken, Adelheid Wiemer-Kruel, Klemens Budde, Klaus Marquard, Markus Knuf, Andreas Hahn, Hans Hartmann, Andreas Merkenschlager, Regina Trollmann.
